# Anesthesia management of a patient with claustrophobia undergoing surgery for rhegmatogenous retinal detachment: A case report

**DOI:** 10.1097/MD.0000000000035624

**Published:** 2023-10-20

**Authors:** Xiang Li, Qiaomei Zhou, Xingan Zhang, Bo Xu

**Affiliations:** a Department of Anesthesiology, General Hospital of Southern Theatre Command of PLA, Guangzhou, China; b Graduate School, Guangzhou University of Chinese Medicine, Guangzhou, China.

**Keywords:** anesthesia induction, anxiety disorder, claustrophobia, panic attacks, phobic disorder

## Abstract

**Introduction:**

Claustrophobia is a form of phobic anxiety disorder characterized by panic attacks. Anesthesia in patients with claustrophobia poses a challenge because these patients reject all treatments in an enclosed space. When such patients are treated in uncomfortably enclosed environments, it can cause mental distress and even sudden psychiatric death.

**Case presentation:**

We report the case of a 55-year-old man with severe anxiety disorder and claustrophobia who required anesthesia for the surgical treatment of rhegmatogenous retinal detachment. This patient had a history of severe anxiety and claustrophobia for more than 40 years, without having received any treatment for the condition. The patient had failed to tolerate multiple chamber surgeries. Following multidisciplinary discussion, the patient’s surgery was performed under general anesthesia in the operating room after the patient underwent induction of anesthesia outside the operating room.

**Conclusions:**

This case report shows that patients with claustrophobia need to be provided a comfortable environment for induction and awakening from anesthesia.

## 1. Introduction

Claustrophobia is a type of phobic anxiety disorder characterized by unpredictable panic attacks. Panic attacks start with fear or terror and may be associated with a sense of impending doom. Patients with claustrophobia experience panic attacks and try to flee when in enclosed spaces. Symptoms during a panic attack include a feeling of suffocation, chest tightness or pain in the chest, and fear of being unable to control themselves. In extreme cases, fainting or sudden death may occur.^[1]^ In claustrophobia, fear of suffocation is a notable symptom. The closed operating room environment can trigger panic attacks in patients with claustrophobia. Therefore, anesthesia management of these patients is extremely challenging. Here, we have described the case of a patient with claustrophobia who underwent successful anesthesia induction and awakening in the operating room hall, indicating that this may be an effective method for anesthesia management in patients with claustrophobia undergoing surgery.

## 2. Case presentation

A 55-year-old man (height, 176 cm; weight, 95 kg) with rhegmatogenous retinal detachment presented to the hospital after experiencing sudden blackout of vision in the right eye for over 20 days prior to his visit. He had a medical history of claustrophobia for over 40 years and severe obstructive sleep apnea syndrome (OSAS); however, he had not undergone any treatment. He had no medical history of chronic systemic diseases such as hypertension, diabetes, coronary heart disease, allergies, or surgery. The preoperative physical examination was unremarkable.

Surgery under local anesthesia was initially planned to take place in an ophthalmic operating room. The first operation failed, as the patient experienced a panic attack upon entering the operation room. The ophthalmologist subsequently applied for anesthesia sedation surgery the next day and consulted the anesthesiology department. After consultation with the anesthesiology department, it was found that the patient had severe OSAS, and thus, the risk of adverse events surrounding surgery under sedation in the ophthalmic operating room was high. As a result, the patient’s safety could not be guaranteed, and general anesthesia surgery was recommended in the anesthesiology operating room. On the day of the surgery, the patient was so resistant to the unfamiliar environment of the anesthesiology operating room that the operation had to be canceled again. After building trust with the patient, the anesthesiologist accompanied the patient to the anesthesiology operating room. However, the patient still experienced a panic attack when he entered the room. He complained of a severe headache, throat obstruction, near suffocation, and an uncontrollable and desperate need to escape the operating room. Neither the nurse nor the doctor could comfort him effectively. Finally, the operation was canceled again. After multidisciplinary consultation, the anesthesiologist recommended that the patient be accompanied by family members for anesthesia sedation outside the operating room of the anesthesiology department, followed by completion of the operation under general anesthesia in the operating room.

The patient underwent routine fasting. On the day of the surgery, the patient, accompanied by his family members, went to the hall outside the operating room of the anesthesiology department. He half-laid on the surgical transfer bed, facing a large window in the hall. The patient felt comfortable looking out of the window, and the anesthesiologist relaxed the patient through conversation. After cannulating an upper extremity peripheral vein, intravenous midazolam (3 mg) and propofol (30 mg) were administered for induction, and the patient was quickly transferred to the operating room after he lost consciousness. He was administered high-flow oxygen via mask for FiO_2_ 100%, oxygen flow 6 L/minutes, with routine monitoring of vital signs. Heart rate was 65 beats per minute, blood pressure 112/55 mm Hg, oxygen saturation 100%, temperature 36.5°C, and bispectral index 75. Intravenous injection of etomidate (20 mg), sufentanil (10 μg), and dexmedetomidine hydrochloride infusion (1 μg/kg during the first 10 minutes) were administered. After stabilization of the heart rate and blood pressure, intravenous injection of cis-atracurium (15 mg) was given to complete anesthesia induction. Oxygen was administered via a mask to assist with breathing, and mechanical ventilation was started after oral placement of a No. 4 laryngeal mask following muscle relaxation. Subsequently, intravenous targeted infusion of propofol (3.0–6.0 μg/mL) and remifentanil (2.0–8.0 ng/mL) and an intravenous dexmedetomidine pump were started for intraoperative maintenance of bispectral index 40 to 60. The surgery took 55 minutes. The operation went smoothly, and the intraoperative vital signs were stable. After the operation, the patient was taken to the hall outside the operating room and kept in a supine position facing the window of the hall. The oxygen bag provided oxygen compound air for spontaneous breathing with a heart rate of 55 beats per minute, blood pressure 96/62 mm Hg, and oxygen saturation 100%. After spontaneous breathing with oxygen saturation ≥ 98% under room air, the laryngeal mask was removed once extubation conditions were reached. The patient had no difficulty in breathing after removing the laryngeal mask, and the semi-recumbent position kept the patient’s airway open and comfortable. After 30 minutes of observation, the patient was fully conscious, breathing was completely restored, and he could communicate normally. He was then sent back to the ward, and all his vital signs remained stable after the operation.

After a few days, the patient was interviewed over the telephone. He had returned to his daily routine, and there were no long-term postoperative adverse events. He described having a fear of enclosed spaces that could be traced back to childhood experiences. He said that he could consciously control his emotions before the surgery, but still experienced uncontrollable fear unconsciously during the operation; however, the extra-operative anesthesia and awakening experience were comfortable.

Written informed consent for publication of this report was obtained from the patient and his family.

## 3. Discussion

Claustrophobia is an uncommon phobic anxiety disorder, and its prevalence varies from 7.7% to 12.5%^[2]^ in various populations. The etiology of claustrophobia is not well understood; studies have shown that it is mainly related to genetic and environmental factors.^[3]^ Our patient seemed to have claustrophobia due to a traumatic childhood experience. He was not afraid of a closed environment per se, but had extreme fear of the panic attacks he experienced in closed environments. He often deliberately avoided small, enclosed, restricted, or trapped places, and could not relax in windowless, enclosed spaces.

Anxiety on the day of the surgery is common; however, patients with extreme anxiety need to be taken seriously, and a psychiatrist should be consulted if possible. A thorough understanding of the patient is required before surgery. Patients with claustrophobia need to cooperate when undergoing clinical diagnosis, treatment, or elective surgery, and it takes a long time to complete professional psychotherapy through preoperative psychology or psychiatry. Therefore, psychotherapy combined with drug sedation is often used for clinical diagnosis, treatment, or elective surgery. Psychiatrists often recommend that conscious patients with claustrophobia be given anesthesia in a restful environment. Hence, anesthesia induction outside the anesthesiology operating room is recommended, and the patient can be sent to the operating room soon after loss of consciousness.

Based on the severity of the anxiety disorder, patients may experience different types of claustrophobic events.^[4]^ The harm caused by the fear of suffocation is extremely serious. Our claustrophobic patient with severe anxiety disorder experienced panic attacks in enclosed spaces. The anesthesiologist placed him outside the operating room, facing a window, while inducing anesthesia. Too-shallow sedation can easily aggravate the patient’s fear and anxiety and even cause adverse consequences such as sudden death. Propofol and dexmedetomidine^[5,6]^ are typically used for anesthesia induction and maintenance in various situations as their administration can be easily controlled, respiratory depression is usually transient, and recovery is typically complete. Therefore, in our case, propofol was used to sedate the patient outside the operating room, and after the patient’s loss of consciousness, he was transferred to the operating room to complete the remaining anesthesia induction steps.

Target-controlled infusion of the short-acting anesthetic agents propofol and remifentanil, combined with dexmedetomidine pumping, were used intraoperatively. Precision anesthesia can effectively ensure adequate sedation and respiratory management during transfer from the intraoperative to the extra-operative period, so that the patient can regain consciousness within the predicted time after arriving in the operating room hall and there are no relevant psychological symptoms associated with the surrounding environment upon awakening. The patient in this report was obese and had severe OSAS, and a key principle of perioperative airway management is to ensure airway patency. In obese patients with OSAS, where mask ventilation or tracheal intubation is not feasible, a laryngeal mask is used as the primary airway support. Short-acting anesthetics can ensure complete recovery of patients with apnea after surgery without any drug-induced apnea. Postoperatively, the patient’s respiratory depression was closely monitored, and when the patient was fully conscious, he was sent to the ward.

Our study had 1 key limitation. We did not initially pay close attention to the level of anxiety the patient was experiencing; therefore, the patient canceled the surgery multiple times due to panic attacks. Cases with claustrophobia, therefore, may require preoperative psychiatric consultation.

## 4. Conclusion

Few reports exist on perioperative anesthesia management in patients with claustrophobia. Our case report demonstrates an effective method for perioperative management of patients with claustrophobia. Further research is still needed, however, on the anesthetic management of such patients.

## Author contributions

**Conceptualization:** Qiaomei Zhou.

**Project administration:** Xiang Li.

**Writing – review & editing:** Xingan Zhang, Bo Xu.

## Corrections

This article was originally published with incorrect information for affiliation a. Affiliation a has now been updated to “Department of Anesthesiology, General Hospital of Southern Theatre Command of PLA, Guangzhou, China.” In addition, authors Qiaomei Zhou, Xingan Zhang, and Bo Xu were published with the incorrect affiliation. Their affiliations have now been updated to affiliation a.
